# Activity of ALK Inhibitors in Renal Cancer with ALK Alterations: A Systematic Review

**DOI:** 10.3390/ijms23073995

**Published:** 2022-04-03

**Authors:** Giovanni Maria Iannantuono, Silvia Riondino, Stefano Sganga, Mario Roselli, Francesco Torino

**Affiliations:** Medical Oncology Unit, Department of Systems Medicine, University of Rome Tor Vergata, Via Montpellier 1, I-00133 Rome, Italy; gmiannantuono@gmail.com (G.M.I.); silvia.riondino@uniroma2.com (S.R.); stefano.sganga@gmail.com (S.S.)

**Keywords:** renal cell carcinoma, ALK, ALK inhibitors, ALK-RCC, systematic review

## Abstract

Renal cell carcinoma (RCC) associated with anaplastic lymphoma kinase (ALK) gene rearrangements (ALK-RCC) is currently considered an “emerging or provisional” tumor entity by the last World Health Organization classification published in 2016. Although several studies assessing ALK-RCC’s clinical and histological characteristics have been published in recent years, only a few publications have evaluated the activity of ALK inhibitors (ALK-i) in this subgroup of patients. Considering the well-recognized efficacy of this evolving class of targeted therapies in other ALK-positive tumors, we conducted a systematic review to evaluate the reported activity of ALK-i in the ALK-RCC subtype. MEDLINE was searched from its inception to 7 January 2022 for case reports and case series on adult metastatic ALK-RCC patients treated with ALK-i whose therapeutic outcomes were available. A virtual cohort of ALK-RCC patients was created. Our results showed a favorable activity of first- and second-generation ALK-i in pretreated ALK-RCC patients in terms of either radiological response or performance status improvement. We hope that the present work will prompt the creation of large, multi-institutional clinical trials to confirm these promising early data.

## 1. Introduction

### 1.1. Rationale

In the last decade, the increasing knowledge in molecular pathology and the genetics of renal cancer has led to the identification of new potential subtypes of renal cell carcinoma (RCC) [1], as demonstrated by the recognition of several “emerging or provisional” renal tumor entities in the “WHO classification of tumors of the urinary system and male genital organs” published in 2016 [2]. However, although these novel subtypes may appear to be distinct tumor entities, further studies on morphology, immunohistochemistry, and molecular biology are needed to define their diagnostic criteria and assess their clinical outcomes [2].

**Figure 1:**
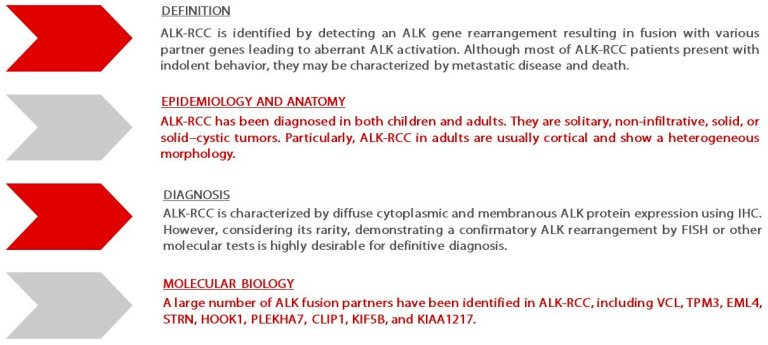
Summary of ALK-RCC features adapted from the “GUPS update on renal neoplasia”—Ref. [6]. Abbreviations: ALK—anaplastic lymphoma kinase; ALK-RCC—renal cell carcinoma associated with ALK gene rearrangements; CLIP1—cytoskeleton-associated proteins-Gly domain containing linker protein 1; EML4—echinoderm microtubule-associated protein-like 4; FISH—fluorescence in situ hybridization; GUPS—Genitourinary Pathology Society; HOOK1—hook microtubule tethering protein 1 kinase; IHC—immunohistochemistry; KIF5B—kinesin family member 5B; PLEKHA7—Pleckstrin homology domain containing A7; STRN—striatin calmodulin-binding protein; TPM3—tropomyosin 3; VCL—vinculin.

RCC associated with anaplastic lymphoma kinase (ALK) gene rearrangements (ALK-RCC) is a novel renal cancer entity, currently considered as “emerging or provisional” [2]. ALK is a receptor protein kinase that plays a physiologic role in the nervous system’s development. It is encoded by the ALK gene located on the 2p23 chromosome [3]. ALK rearrangements with other fusion partners have been described in various malignancies, such as anaplastic large-cell lymphoma (ALCL), non-small cell lung cancer (NSCLC), anaplastic thyroid carcinoma, and others [4]. ALK-RCC accounts for less than 1% of all RCC subtypes [5]. However, a growing number of publications assessing the clinical presentation and histological characteristics of patients affected by ALK-RCC have been published in recent years (Figure 1) [1,6], mainly as case reports and case series [5].

Nowadays, the development of ALK inhibitors (ALK-i) has changed the treatment landscape of cancer patients with ALK alterations, as demonstrated by the dramatic and often prolonged responses to treatment achieved in patients affected by metastatic NSCLC [7]. In contrast, the activity of ALK-i in ALK-RCC patients has been reported in only a few publications. To the best of our knowledge, the impact of ALK-i on this population has never been reviewed.

### 1.2. Objectives

We conducted a systematic review according to the preferred reporting items for systematic reviews and meta-analyses (PRISMA) guidelines (see Supplementary Material A) [8], aiming to evaluate the reported activity of ALK-i in ALK-RCC patients published in the scientific literature.

## 2. Methods

### 2.1. Eligibility Criteria

We included case reports and case series on adult patients affected by ALK-RCC who were treated with ALK-i. Only papers written in English or European languages were considered. The case series had to provide single descriptions of the reported cases to be included in the review. Letters to the editor providing single-case descriptions were also eligible if all the previous criteria were satisfied. All the references of the included studies were later hand-searched for additional eligible publications to include. On the contrary, case reports and case series on patients affected by ALK-RCC not treated with ALK-i were excluded, as well as narrative or systematic reviews on this topic.

### 2.2. Information Sources

We searched the electronic PubMed database from its inception to 7 January 2022 to identify all relevant papers. No research filters for case reports/case series or language limits were applied. All the results derived from the literature search were uploaded to a reference management software.

### 2.3. Search Strategy

The search strategy for eligible publications was established through a discussion among the authors. The following syntax was used: “(ALK OR ALK-rearranged) AND (renal cancer OR kidney cancer OR renal cell carcinoma* OR renal cell cancer OR renal)”.

### 2.4. Selection Process

The literature search was conducted independently by two of the authors. A two-stage study selection process was used. Firstly, all titles and abstracts were initially screened for potential relevance. Secondly, full texts of results deemed eligible were retrieved and further assessed for eligibility. Agreement of both authors was required for exclusion at both stages. A consultation with a third author was required to resolve disagreements on study selection by consensus. The authors achieved a complete consensus on publications to include in the systematic review before starting with data collection.

### 2.5. Data Collection Process

Two authors developed a data-charting template using Microsoft Excel to extract the variables of the included studies. They independently extracted the data and discussed the results in an interactive process. A third author verified all the extracted data. 

### 2.6. Data Items

The variables extracted from the included papers were divided into three groups: study characteristics, patient characteristics, and exposure characteristics. The variables of the first group were: first author, publication date, journal of publication, type of study, and number of patients reported. The variables of the second group were: age, gender, presenting symptoms and signs, tumor staging, histotype of RCC, metastatic sites, type of ALK alteration, and previous surgical and medical treatments. Lastly, the variables of the third group were: type of ALK-i with the related therapeutic outcome described in terms of either best response according to the response evaluation criteria in solid tumors (RECIST) or survival (if available).

### 2.7. Study Risk of Bias Assessment

A tool proposed by Murad et al. to evaluate the methodological quality of case series and case reports was used to assess the risk of bias of publications included in the systematic review [9]. This tool considers eight different questions categorized in four domains: selection, ascertainment, causality, and reporting. A binary response (yes = 1 and no = 0) was assigned to every question and then an aggregate score was formulated for every publication [9].

### 2.8. Effect Measures

Variables extracted from eligible publications were described using numbers and proportions for categorical variables, and mean, standard deviation, median, and interquartile range for continuous variables.

### 2.9. Synthesis Methods

We reported aggregated data obtained from the included studies. Due to the limited sample size, no inferential or predictive statistics analyses were performed.

## 3. Results

### 3.1. Study Selection

**Figure 2:**
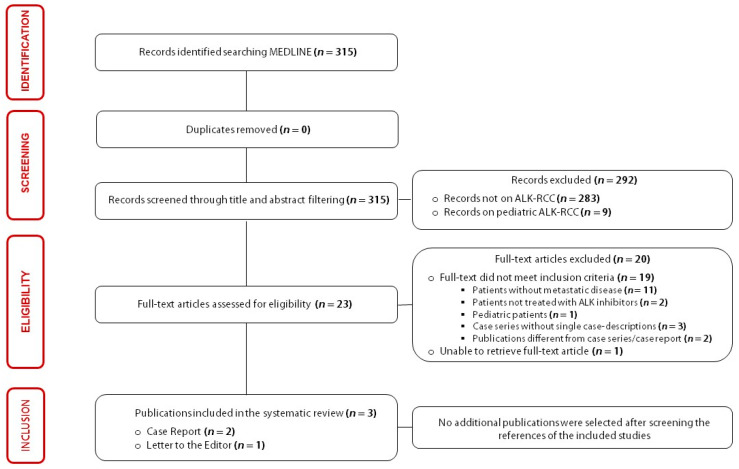
Flowchart of the results of literature search and identification process of included publications.

The literature search results and the identification process of papers included in the present systematic review are summarized using a PRISMA flow diagram in Figure 2.

### 3.2. Study Characteristics

Three papers, published between 2018 and 2021, were included in this systematic review: two case reports and one letter to the editor. All the publications were written in English.

### 3.3. Results of Individual Studies

The results of single sources of evidence eligible for the present systematic review are described in Table 1.

### 3.4. Results of Syntheses

Data extracted from the selected papers allowed us to create a cohort of five patients affected by ALK-RCC treated with ALK-i, with available therapeutic outcomes (Table 2). 

The median age of the patients was 44 years (range: 30–85 years). The most frequent initial symptoms were a palpable mass in 40% of patients, respiratory symptoms (40%), back pain (20%), hematuria (20%), and weight loss (20%). Only one patient (20%) was asymptomatic at the time of diagnosis. All patients were affected by advanced RCC. The most common metastatic sites were lungs (80%), lymph nodes (60%), bones (20%), brain (20%), and thyroid (20%). The histological diagnosis was papillary RCC (PRCC) in 80% of cases and mixed histology RCC in 20% of patients. ALK alterations were detected with different procedures either in neoplastic tissue provided by biopsies of distant RCC metastases or circulating tumor DNA (ctDNA). In the case report published by Varchetta et al. [12], a genomic profiling test performed on a cell-block obtained by a lymph-node fine needle biopsy showed a pathogenic rearrangement (c.3512T > A) in the ALK gene. In the case report described by Zhou et al. [11], a rearrangement of the ALK gene with an unknown fusion partner was detected on a lung metastasis tissue through a FISH test performed due to ALK-positive results at the immunohistochemical analysis. In contrast, Pal et al. [10] detected in three patients a translocation between echinoderm microtubule-associated protein-like 4 (EML4) and ALK through a genomic profiling test performed on ctDNA. All patients received one or multiple lines of treatment before the administration of ALK-i: sunitinib in 40% of cases, savolitinib (40%), bevacizumab (20%), cabozantinib (20%), chemotherapy (20%), everolimus (20%), nivolumab (20%), pazopanib (20%), sorafenib (20%), and temsirolimus (20%). ALK-i administered to patients were alectinib in 80% of cases and crizotinib in 20% of cases. Concerning the assessment of ALK-i activity, 80% of patients achieved a radiological “partial response”, and 20% of patients were considered to have a “stable disease”. No patients were reported to have a progressive disease during ALK-i treatment. The mean progression-free survival (PFS) of ALK-i treated patients was 4.8 months (range: 3–9 months), despite the treatment still being ongoing in most cases at the time of publication.

### 3.5. Reporting Biases

We evaluated all the publications included in the review with the tool proposed by Murad et al. [9], assigning an aggregate score to every study (see Supplementary Material B).

## 4. Discussion

**Figure 3:**
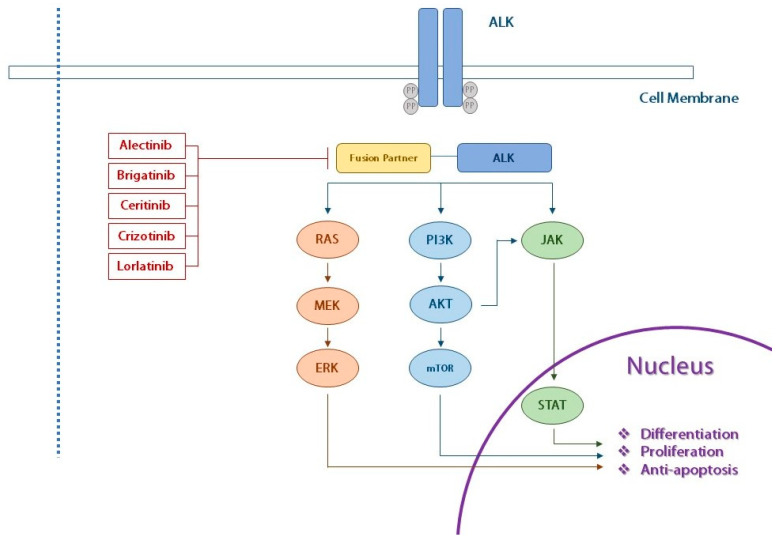
Illustration of ALK fusion oncogene with main downstream signaling pathways. Abbreviations: ALK—anaplastic lymphoma kinase; ERK—extracellular signal-regulated kinase; JAK—Janus kinase; mTOR—mammalian target of rapamycin; MEK—mitogen-activated protein kinase; PI3K—phosphatidylinositol 3-kinase; PP—pyrophosphate; AKT—protein kinase B; RAS—rat sarcoma virus; STAT—signal transducer and activator of transcription.

In recent years, the increasing understanding of RCC genetics has led to the identification of potential new renal tumor entities [1], classified as “emerging or provisional” in the recent “WHO classification of tumors of the urinary system and male genital organs”, including the ALK-RCC subtype [2]. ALK is a receptor protein kinase belonging to the superfamily of insulin receptors and encoded by the ALK gene located on the 2p23 chromosome [3]. The role of ALK in tumorigenesis was initially shown in ACLC by Morris et al. in 1994 [13]. Several mechanisms may cause ALK hyperactivation, such as translocations involving the kinase domain of the protein, point mutations, and gene amplification resulting in the pathological activation of pathways contributing to the cell acquisition of malignant phenotypes [3]. In particular, the phosphorylation of ALK results in the activation of downstream signaling pathways which can promote cell proliferation and differentiation, such as the Janus kinase (JAK)/signal transducer and activator of transcription (STAT), phosphatidylinositol-3-kinase (PI3K)/protein kinase-B (AKT)/mammalian target of rapamycin (mTOR), and rat sarcoma virus (RAS)/mitogen-activated protein kinase (MAPK) pathways (Figure 3) [14,15]. 

Nowadays, different ALK alterations have been detected in several solid tumors such as ACLC, inflammatory myofibroblastic tumors, neuroblastomas, and NSCLC [4]. In parallel, the identification of ALK-positive tumors prompted the creation of a new class of targeted therapy, named ALK-i. Crizotinib was the first ALK-i drug approved in the setting of metastatic NSCLC. Subsequently, second-generation ALK-i (alectinib, brigatinib, and ceritinib) and, recently, a third-generation ALK-i (lorlatinib) have been introduced in the same clinical setting [16]. All these agents have shown an undeniable efficacy in patients affected by ALK-positive advanced NSCLC, thus revolutionizing the treatment landscape in this subgroup of patients [17].

Despite a lack of encouraging results, the activity of crizotinib has already been evaluated in the field of RCC treatment in recent years, due to its ability to inhibit the mitogen-activated protein kinase (MET). Indeed, an increased expression of MET has been implicated in either tumor angiogenesis or the development of resistance to anti-vascular endothelial growth factor (VEGF) therapies [18]. In 2019, Michaelson et al. published the results of a phase Ib study aimed to evaluate the combination of axitinib and crizotinib in advanced solid tumors and metastatic RCC, showing a manageable safety profile and evidence of modest antitumor activity for this combination [19]. Concomitantly, the genomic characterization of PRCC led to the identification of molecular alterations in the MET gene [20], suggesting the opportunity to investigate the effects of the MET inhibitors (including crizotinib) also in this RCC subtype. In 2017, Schoffski et al. published the results of a single cohort of patients affected by advanced PRCC treated with crizotinib enrolled in the CREATE trial [21]. Patients were divided into two subgroups depending on the presence of MET alterations. Only a small proportion of patients achieved durable responses, independent from the presence of MET alterations [21]. In 2021, Pal SK et al. published the results of the SWOG 1500 trial, which compared the standard of care (SoC), represented by sunitinib, with the other MET kinase inhibitors, including crizotinib, in metastatic PRCC patients [22]. The assignment to the crizotinib group was halted after a prespecified futility analysis. The final results showed that cabozantinib determined a significantly longer PFS in comparison to SoC and other MET inhibitors [22].

**Figure 4:**
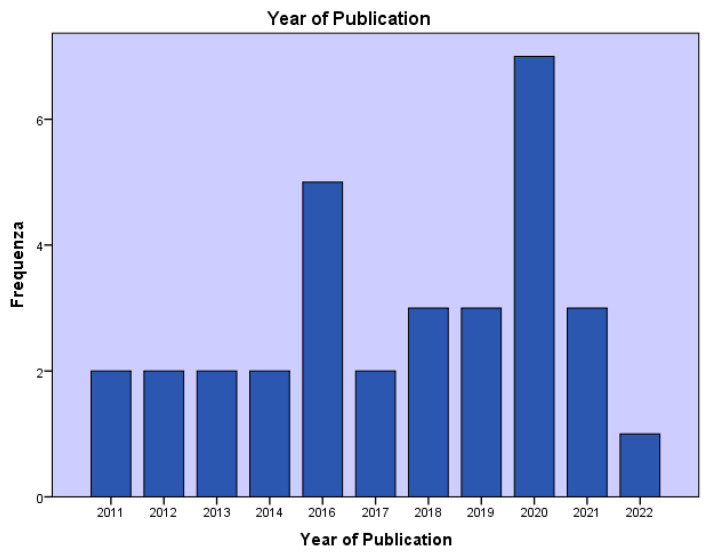
Number of publications on ALK-RCC based on the year of publication.

ALK-RCC accounts for less than 1% of all renal neoplasms [5]. It was described for the first time in 2010 [23]. Since then, a growing number of publications assessing the clinical presentation and histological characteristics of patients affected by ALK-RCC have been published (Figure 4) [6]. Indeed, approximately forty ALK-RCC cases have been reported (Table 3) in both adults and children, aged between 6 and 85 years [24]. Although most of the ALK-RCC patients present with an indolent behavior, they may be characterized by metastatic disease and death, as documented in about 25% of reported cases [6]. ALK-RCC are solitary tumors, generally not associated with any syndrome [25]. Concerning the gross pathological features, they are solid or solid–cystic tumors showing white-grey to yellow and variegated cut surfaces and can originate either from the medulla or the cortex [26]. The most frequent translocations reported in ALK-RCC patients are the fusion of the vinculin (VCL) and ALK, typically in young patients with the sickle cell trait [27], the TPM3–ALK and EML4–ALK fusions in tumors characterized by papillary, solid, and mucinous cribriform components. Furthermore, striatin (STRN) and ALK translocation typically appear in tumors with papillary, solid, tubular, and mucinous cribriform structures associated with psammoma bodies [28]. 

In addition, a few retrospective studies described ALK-RCC incidence in different populations. In 2012, Sukov et al. reported the results of the first single-institution retrospective study aiming to evaluate the frequency of ALK alterations in a large series of American adult patients affected by RCC. A cohort of 534 patients was analyzed, and a rearrangement of the ALK gene was detected in 2 patients (<1% of all patients), both affected by papillary RCC [31]. In the same year, Sugawara et al. described the results of a retrospective screening for ALK alterations of 355 renal tumors from Japanese patients, with the detection of two positive patients [30]. In 2013, Lee et al. reported the analysis’ results of a cohort of 829 resected RCC from Korean patients. Only one patient was diagnosed with ALK-RCC [32]. Three years later, Yu et al. reported data obtained through screening 477 resected RCC in the Chinese population, identifying only two patients with ALK alterations [40]. In 2019, Gorczynski et al. published the results of the analysis of 1019 renal tumors from Polish patients to evaluate ALK-RCC incidence, without any positive cases identified [47]. 

As a result, considering the growing attention to ALK-RCC and the well-recognized benefit of ALK-i in other ALK-positive tumors [48], we aimed to systematically revise the available literature to evaluate the activity of ALK-i in ALK-RCC patients. According to the inclusion criteria of the systematic review, only three publications were included that reported a single-case description of clinical and pathological features of adult ALK-RCC patients treated with ALK-inhibitors. Firstly, in 2018 Pal et al. showed the therapeutic outcomes of three advanced renal cancer patients treated with alectinib, a second-generation ALK-i [10]. All patients received multiple lines of treatment with different anticancer agents, including multi-target tyrosine kinase inhibitors (TKI) and immune checkpoint inhibitors (ICI). After being detected with ALK-EML4 translocations on ctDNA through a genomic profiling test, they started the treatment with alectinib. All patients achieved a radiological partial response with a concurrent improvement of performance status [10]. Secondly, in 2020, Zhou et al. reported the case of a young woman who had a progressive disease with pulmonary metastases after nephrectomy for a localized RCC [11]. The patient started a first-line treatment with sunitinib. After detecting an immunohistochemical ALK positivity, confirmed by a FISH test, the patient switched to a systemic therapy with crizotinib, achieving a radiological stable disease [11]. Thirdly, in 2021, Varchetta et al. described the case of a patient with relapsed renal cancer who received multiple lines of treatment, except for ICI due to a pre-existing severe rheumatic disease. After detecting an ALK rearrangement on neoplastic tissue, the patient started alectinib, achieving a radiological partial response [12]. Lastly, Thorner et al. reported the case of a twelve-year-old patient with a relapsed ALK-RCC who received an unspecified ALK-i for more than one year. The patient was still continuing the treatment at the time of publication [34]. However, the publication was not included in the review due to the pediatric age of the patient.

Despite the small number of publications available on ALK-RCC treated with ALK-i, several issues deserve to be discussed. Firstly, all ALK-RCC patients treated with ALK-i achieved a radiological response associated with improved performance status, although they all received previous systemic therapies [10,11,12]. Considering that most of the anticancer agents used today for the treatment of metastatic RCC are either targeted therapies directed at molecules involved in angiogenesis or ICI [49], promising results in terms of activity for ALK-i could open the way to a new class of anticancer agents for the treatment of patients affected by metastatic RCC. Four patients were treated with alectinib [10,12], a second-generation ALK-i, while only one patient received crizotinib [11]. In addition, considering the recent data on the efficacy of lorlatinib even in controlling the distant metastases localized in the central nervous system [50], it would have been interesting to evaluate its activity in ALK-RCC patients either after progression on a previous ALK-i or as a first-line treatment. In this direction, further promising results were published by Tao et al. [51]. They described the case report of an ALK-RCC patient who relapsed after complete resection of the tumor and, therefore, received first-line treatment with entrectinib, a selective TKI towards neurotrophic tyrosine receptor kinase (NTRK), ROS1, and ALK fusion. The patient maintained an excellent performance status throughout the treatment with a PFS of nineteen months [51].

Secondly, further questions are open on the procedures used to detect ALK alterations and their timing, considering the promising results provided by ALK-i. According to the International Society of Urological Pathology (ISUP), immunohistochemical screening with ALK antibody and the subsequent confirmation by fluorescence in situ hybridization (FISH) or sequencing methods is generally recommended [1]. In the case report published by Zhou et al., ALK rearrangement was detected on neoplastic tissue obtained by a biopsy of a lung metastasis through a FISH test performed after a positive result at the immunohistochemical analysis [11]. In the case report published by Varchetta et al., the ALK alteration was also detected on distant metastases through a genomic profiling test [12]. In contrast, in the three cases reported by Pal et al., the ALK-EML4 translocation was detected in ctDNA through a genomic profiling test, which showed the presence of ALK alterations associated with other mutations, not eligible for other targeted therapies [10]. ALK alterations in all these patients were not detected in specimens from primary RCC but only from distant metastases or ctDNA [10]. In this scenario, the available data on the concordance of ALK rearrangements between the primary lung tumor and metastases are encouraging. Indeed, in 2017 Hou et al. published a single-center study aimed to investigate the concordance of ALK rearrangements between lung adenocarcinoma and the paired metastatic lymph nodes [52]. The results showed a concordance rate of 98%, concluding that specimens provided from the primary tumor or the lymph-node metastases were equally suitable for ALK rearrangements’ detection [52]. These results were confirmed by a study published in the same year by Ma et al., where a high concordance of ALK status was found between the primary tumor and corresponding lymph node metastases [53]. Furthermore, considering that approximately 30% of tumor biopsy samples may be insufficient to perform ALK characterization, promising data are available on the role of circulating tumor cells as a surrogate to tissue for the detection of ALK alterations [54]. 

Lastly, these emerging data confirm that ALK is a compelling therapeutic target, as demonstrated by its role as an oncogenic driver in several tumor types of different lineages, including RCC. ALK fusions are extraordinarily versatile oncoproteins that can be targeted with several anticancer agents with a well-recognized efficacy [55]. Furthermore, in the “precision medicine” era where tumor-agnostic therapies represent a new revolutionary approach to cancer treatment [56], ALK-i are earning a potential role in the agnostic-setting of ALK-positive tumors. In this direction, it will be essential to know the final results of two ongoing trials, the CREATE trial [57] and the Alpha-T trial [58]. The former is a phase 2 trial designed to evaluate the antitumor activity of crizotinib across predefined tumor types in patients whose tumors are harboring specific alterations in ALK and/or MET [57]. The latter is a phase 2 trial aimed to evaluate the efficacy and safety of alectinib in participants with ALK-positive locally advanced or metastatic solid tumors other than lung cancer [58].

### 4.1. Future Directions

In the last two decades, the therapeutic scenario of metastatic RCC has been revolutionized, as evidenced by the dramatic improvement of the median survival of RCC patients from less than one year in the 1990s to over four years in the most recent trials [59]. The treatment landscape has evolved from interleukin 2 and interferon-alfa to targeted therapies such as vascular endothelial growth factor receptor inhibitors, mammalian target of rapamycin inhibitors, and ICI [49]. Nevertheless, a considerable effort is still necessary to transform the evolving knowledge in identifying novel RCC entities into more personalized treatments. Indeed, a greater understanding of RCC underlying biological processes is essential to develop future therapeutic agents [60]. For example, the detection of novel fusion genes involved in the development, growth, and survival of cancer cells could lead to the identification of new potential druggable targets for “precision medicine” approaches [61].

In this direction, initial evidence suggests that ALK-RCC patients might benefit from the treatment with ALK-i [60]. Nevertheless, it will be essential to evaluate the activity of ALK-i firstly in large, multi-institutional clinical trials for pretreated ALK-RCC patients. Once the activity of ALK-i has been demonstrated in pretreated patients, it would be of great clinical interest to test their efficacy against the available treatments in this subtype of patients. Indeed, the presence of ALK alterations could allow them to benefit from an evolving class of anticancer agents that have already demonstrated their efficacy in other tumor types.

In parallel, considering the potential efficacy of ALK-i in this RCC subtype, further data are necessary to describe the clinical and histological characteristics of ALK-RCC patients clearly. This would be important to select those patients who can benefit from molecular testing for ALK, in light of the cost and time-consuming procedures to screen all RCC patients at the diagnosis. According to the aforementioned larger case series, PRCC patients are more frequently carriers of ALK alterations than clear-cell RCC patients [30,31,32,40,47]. In addition, ALK testing represents a way to better classify some patients affected by RCC with unclassified histology (uRCC) that have no standard therapy. Indeed, the screening for ALK alterations in uRCC patients would be essential to allow these patients to be treated with a personalized treatment [62]. Lastly, the available data highlight heterogeneity in molecular testing for ALK alterations that could represent one of the explanations for the differences in ALK-RCC rates provided by the larger series published in recent years [62]. In this light, a consensus paper would be useful to provide recommendations for a test algorithm for renal cancer and the quality of the respective test approaches, like the one released regarding ALK testing in NSCLC [63].

### 4.2. Limitations

The main limitation of this systematic review lies in the extraction of data from case reports and case series. These two types of publications are at an increased risk of bias due to their intrinsic nature [9]. As a result, we decided to include case reports and case series in which a single case description was available.

### 4.3. Conclusions

Although ALK-RCC is currently considered an “emerging or provisional” entity by the last WHO classification [2], several studies have been published in recent years assessing the clinical presentation and histological characteristics of ALK-RCC patients. In contrast, only a few publications have evaluated the activity of ALK-i in this subgroup of patients. By systematically revising the available literature, we demonstrated for the first time, to our knowledge, a favorable activity of first- and second-generation ALK-i in pretreated ALK-RCC patients in terms of either radiological response or performance status improvement. We hope that the present work will prompt the creation of large, multi-institutional clinical trials to confirm these promising early data to allow this subgroup of patients to benefit from a rapidly evolving class of targeted therapy that has already demonstrated its efficacy in other tumors.

## 5. Other Information

### 5.1. Registration and Protocol

The protocol was designed a priori and approved by all the authors. In addition, it was registered on the open science framework website https://osf.io/rx9hj/ (accessed on 23 February 2021).

### 5.2. Support

This research did not receive any grant from funding agencies in the public, commercial, or not-for-profit sectors.

### 5.3. Competing Interests

The authors report no declarations of interest.

## Figures and Tables

**Table 1:** Variables extracted from included publications.

Study Characteristics				Patient Characteristics						Exposure Characteristics					
First Author (Year of Publication)	Journal	Type of Study	Number of Cases	Age (Gender)	Initial Symptoms	Tumor Staging	Metastatic Sites	Hystotipe	ALK Alteration	Surgery	First-Line	Second-Line	Subsequent Lines	ALK-i	Outcome
Pal et al. (2018) [10]	Eur Urol.	LE	3	66 (M)	Haematuria	Advanced	Brain—lung	ccRCC-pRCC	EML4-ALK	RN	Pazopanib	Savolitinib	Everolimus—nivolumab—cabozantnib	Alectinib	PR (9 mos)—AwD
-	-	-	-	30 (F)	Excruciating hip—back pain	Advanced	Bone—lung—nodes	pRCC	EML4-ALK	RN	Savolitinib	-	-	Alectinib	PR (4 mos)—AwD
-	-	-	-	85 (F)	Shortness of breath	Advanced	Lung	pRCC	EML4-ALK	No	CBDCA-Tax	-	-	Alectinib	PR (4 mos)—AwD
Zhou et al. (2020) [11]	Transl Androl Urol.	CR	1	36 (F)	Follow up	Advanced	Nodes—lung	pRCC	ALK fusion partner not available	RN	Sunitinib	-	-	Crizotinib	PR (3 mos)—AwD
Varchetta et al. (2021) [12]	Recenti Prog Med.	CR	1	44 (F)	NA	Advanced	Nodes—thyroid	NA	c.3512T > A	RN	Sunitinib	Sorafenib	Cabozantinib—bevacizumab—temsirolimus	Alectinib	PR (4 mos)—AwD

Abbreviations: AwD—alive with disease; CBDCA—carboplatin; CR—case report; ccRCC—clear cell renal cell carcinoma; F—female; LE—letter to the editor; M—male; Mos—months; NA—not available; Tax—paclitaxel; pRCC—papillary renal cell carcinoma; PR—partial response; RN—radical nephrectomy.

**Table 2:** Results of quantitative analysis of variables extracted by included publications.

Cohort		Staging		Treatments	
Number of patients	5	**Presence of metastases at diagnosis**		**Treatments before ALK-inhibitors**	
Median age (Range)	44 (30—85)	Bone	1 (20%)	Bevacizumab	1 (20%)
		Brain	1 (20%)	Cabozantinib	1 (20%)
**Clinical presentation**		Lymph-Nodes	3 (60%)	Chemotherapy	1 (20%)
		Lung	4 (80%)	Everolimus	1 (20%)
**Initial symptoms**		Thyroid	1 (20%)	Nivolumab	1 (20%)
Back pain	1 (20%)			Pazopanib	1 (20%)
Hematuria	1 (20%)	**Histology and ALK alterations**		Savolitinib	2 (40%)
Palpable mass	2 (40%)			Sorafenib	1 (20%)
Respiratory symptoms	2 (40%)	**Histological diagnosis**		Sunitinib	2 (40%)
Weight loss	1 (20%)	Papillary	4 (80%)	Temsirolimus	1 (20%)
No symptoms	1 (20%)	Mixed Histology	1 (20%)	**Treatments with ALK-inhibitors**	
		**ALK alterations**		Alectinib	4 (80%)
		ALK-EML4	3 (60%)	Crizotinib	1 (20%)
		c.3512T > A	1 (20%)		
		Fusion partner not specified	1 (20%)	**Outcome**	
				Partial disease	4 (80%)
				Stable disease	1 (20%)

**Table 3:** Previously published cases of ALK-RCC.

First Author	Journal	Year of Publication	Type of Study (Number of Cases)	Age (Gender)	Advanced Stage/Relapsed	Metastatic Sites	Hystotipe	ALK Translocation	Surgery	Medical Therapy
Debelenko et al. [23]	Mod Pathol.	2011	CS (6)	16 (M)	No	Nodes	uRCC	VCL–ALK	RN	No
-	-	2011	-	10 (F)	Yes	Nodes	RMC	VCL–ALK	RN	ChT
-	-	2011	-	12 (M)	Yes	Lung	RMC	VCL–ALK	NA	NA
-	-	2011	-	7 (F)	Yes	Lung	RPC	VCL–ALK	NA	NA
-	-	2011	-	14 (M)	NA	-	RPC	VCL–ALK	NA	NA
-	-	2011	-	6 (F)	No	-	uRCC	VCL–ALK	NA	NA
Mariño-Enríquez et al. [29]	Genes Chromosomes Cancer	2011	CR (1)	6 (M)	No	-	RMC	VCL-ALK	RN	No
Sugawara et al. [30]	Cancer	2012	CS (2)	36 (F)	No	-	uRCC	TPM3-ALK	RN	No
-	-	2012	-	53 (F)	No	-	pRCC—ccRCC	EML4-ALK	RN	No
Sukov et al. [31]	Mod Pathol.	2012	CS (2)	61 (M)	No	-	pRCC	NA	RN	No
-	-	2012	-	59 (M)	No	-	pRCC	NA	RN	No
Lee C et al. [32]	Korean J Pathol.	2013	CR (1)	44 (M)	No	-	RCC	NA	RN	No
Smith et al. [27]	Am J Surg Pathol.	2014	CR (1)	6 (M)	No	-	RCC	VCL-ALK	RN	No
Ryan et al. [33]	Virchows Arch.	2014	CR (1)	36 (M)	No	-	RCC	NA	RN	No
Kusano et al. [28]	Am J Surg Pathol.	2016	CS (2)	33 (F)	Yes	Nodes	pRCC	STRN-ALK	RN	NA
-	-	2016	-	38 (M)	Yes	Nodes—Liver	RCC	STRN-ALK	RN	Sunit.
Thorner et al. [34]	Pathol Res Pract.	2016	CR (1)	12 (F)	Yes	Paraspinal mass—Soft tissue lesion	RCC	TPM3-ALK	RN	ALK-i
Cajaiba et al. [35]	Genes Chromosomes Cancer	2016	CR (1)	16 (M)	NA	-	RCC	TPM3-ALK	NA	NA
-	-	2016	-	6 (M)	NA	-	pRCC	VCL-ALK	NA	NA
-	-	2016	-	16 (M)	NA	-	pRCC	VCL-ALK	NA	NA
-	-	2016	-	6 (M)	NA	-	pRCC	VCL-ALK	NA	NA
-	-	2016	-	16 (F)	NA	-	RCC	TPM3-ALK	NA	NA
-	-	2016	-	14 (M)	NA	-	RCC	TPM3-ALK	NA	NA
Jeanneau et al. [36]	Pathol Res Pract.	2016	CR (1)	40 (F)	No	-	RCC	VCL-ALK	RN	No
Cajaiba et al. [37]	Genes Chromosomes Cancer	2016	CR (1)	16 (M)	No	-	pRCC	HOOK1-ALK	RN	NA
Yu et al. [38]	Histopathology	2017	CS (2)	49 (M)	No	-	uRCC	TPM3-ALK	RN	No
-	-	2017	-	52 (F)	No	-	pRCC	EML4-ALK	RN	No
Oyama et al. [39]	Pathol Int.	2017	CR (1)	19 (F)	No	-	RCC	NA	RN	No
Bodokh et al. [40]	Cancer Genet.	2018	CR (1)	55 (F)	No	-	RCC	TPM3-ALK	RN	No
Yang et al. [41]	Diagn Pathol.	2019	CR (1)	58 (M)	No	-	RCC	TPM3-ALK	RN	No
Chen et al. [42]	Oncol Rep.	2020	CS (2)	38 (M)	No	-	ccRCC	EML4-ALK	RN	No
-	-	2020	-	59 (M)	NA	-	ccRCC	EML4-ALK	RN	NA
Woo et al. [43]	Yonsei Med J.	2020	CR (1)	14 (M)	No	-	RCC	TPM3-ALK	RN	No
Hang et al. [44]	Virchows Archiv.	2020	CR (1)	68 (F)	No	-	MA-like	PLEKHA7-ALK	PN	No
Zhu et al. [45]	Pathol Res Pract.	2020	CR (1)	15 (F)	NA	-	pRCC	HOOK1-ALK	RN	NA
Kuroda et al. [46]	Mod Pathol.	2020	CS (12)	33 (F)	No	-	uRCC	CLIP1-ALK	RN	No
-	-	2020	-	51 (F)	No	-	MTSC-RCC	KIF5B-ALK	RN	No
-	-	2020	-	25 (F)	No	-	MA-like	STRN-ALK	PN	No
-	-	2020	-	48 (F)	No	-	RCC	STRN-ALK	PN	No
-	-	2020	-	54 (M)	No	-	uRCC	TPM3-ALK	PN	No
-	-	2020	-	56 (M)	No	-	uRCC	EML4-ALK	RN	No
-	-	2020	-	42 (M)	No	-	RCC	STRN-ALK	RN	No
-	-	2020	-	58 (F)	No	-	uRCC	TPM3-ALK	RN	No
-	-	2020	-	43 (M)	No	-	RCC	KIAA1217-ALK	RN	No
-	-	2020	-	40 (F)	No	-	RCC	EML4-ALK	PN	No
-	-	2020	-	38 (M)	No	-	uRCC	TPM3-ALK	PN	No
-	-	2020	-	68 (F)	No	-	MA-like	PLEKHA7-ALK	PN	No

Abbreviations: ALK-i—ALK inhibitor; CR—case report; CS—case series; ChT—chemotherapy; ccRCC—clear cell renal cell carcinoma; CLIP1—cytoskeleton-associated proteins-Gly domain containing linker protein 1; EML—echinoderm microtubule-associated protein-like 4; F—female; HOOK1—hook microtubule tethering protein 1 kinase; KIF5B—kinesin family member 5B; M—male; MA-like—metanephric carcinoma-like; MTSC-RCC—mucinous tubular and spindle renal cell carcinoma; NA—not available; pRCC—papillary renal cell carcinoma; PN—partial nephrectomy; PLEKHA7—Pleckstrin homology domain containing A7; RN—radical nephrectomy; RCC—renal cell carcinoma; RMC—renal medullary carcinoma; Sunit.—sunitinib; STRN—striatin calmodulin-binding protein; TPM3—tropomyosin 3; uRCC—unclassified renal cell carcinoma; VCL—vinculin.

## Data Availability

Not applicable.

## Supplementary Materials

The following are available online at https://www.mdpi.com/article/10.3390/ijms23073995/s1.
