# Supplementary material for: Activity of ALK Inhibitors in Renal Cancer with ALK Alterations: A Systematic Review
Source: Int J Mol Sci. 2022 Apr 3;23(7):3995. doi: 10.3390/ijms23073995 (PMC8999731; doi:10.3390/ijms23073995)
Supplement: Supplementary file 1 [file ijms-23-03995-s001.zip › Supplementary/Supplementary Material B.pdf]

Varchetta, V.; Campanella, C.; Rossi, M.; Verzaro, R.; Vitale, M.; Soda, G.; Mancuso, A. Identification of Anaplastic Lymphoma Kinase Fusion in Clear Cell Renal Carcinoma (ALK-TRCC): A Precision Oncology Medicine Case Report. Recenti Prog Med 2021, 112, 14e–15e, doi:10.1701/3525.35133

**Tool for evaluating the methodological quality of case reports and case series**<br>[Murad et al. BMJ Evid Based Med. 2018]		
**Domains**	**Leading explanatory questions**	**Answer**
Selection	1. Does the patient(s) represent(s) the whole experience of the investigator (centre) or is the selection method unclear to the extent that other patients with similar presentation may not have been reported?	0
Ascertainment	2. Was the exposure adequately ascertained?	1
	3. Was the outcome adequately ascertained?	1
Causality	4. Were other alternative causes that may explain the observation ruled out?	1
	5. Was there a challenge/rechallenge phenomenon?	0
	6. Was there a dose–response effect?	0
	7. Was follow-up long enough for outcomes to occur?	1
Reporting	8. Is the case(s) described with sufficient details to allow other investigators to replicate the research or to allow practitioners make inferences related to their own practice?	1
		5

Zhou, S.; Sun, G.; Wang, J.; Zhang, H. Anaplastic Lymphoma Kinase (ALK) Rearrangement in Adult Renal Cell Carcinoma with Lung Metastasis: A Case Report and Literature Review. Transl Androl Urol 2020, 9, 2855–2861, doi:10.21037/tau-20-1343.

**Tool for evaluating the methodological quality of case reports and case series**<br>[Murad et al. BMJ Evid Based Med. 2018]		
**Domains**	**Leading explanatory questions**	**Answer**
Selection	1. Does the patient(s) represent(s) the whole experience of the investigator (centre) or is the selection method unclear to the extent that other patients with similar presentation may not have been reported?	0
Ascertainment	2. Was the exposure adequately ascertained?	1
	3. Was the outcome adequately ascertained?	1
Causality	4. Were other alternative causes that may explain the observation ruled out?	1
	5. Was there a challenge/rechallenge phenomenon?	0
	6. Was there a dose–response effect?	0
	7. Was follow-up long enough for outcomes to occur?	1
Reporting	8. Is the case(s) described with sufficient details to allow other investigators to replicate the research or to allow practitioners make inferences related to their own practice?	1
		5

Pal, S.K.; Bergerot, P.; Dizman, N.; Bergerot, C.; Adashek, J.; Madison, R.; Chung, J.H.; Ali, S.M.; Jones, J.O.; Salgia, R. Responses to Alectinib in ALK-Rearranged Papillary Renal Cell Carcinoma. Eur Urol 2018, 74, 124–128, doi:10.1016/j.eururo.2018.03.032.

**Tool for evaluating the methodological quality of case reports and case series**<br>[Murad et al. BMJ Evid Based Med. 2018]		
**Domains**	**Leading explanatory questions**	**Answer**
Selection	1. Does the patient(s) represent(s) the whole experience of the investigator (centre) or is the selection method unclear to the extent that other patients with similar presentation may not have been reported?	0
Ascertainment	2. Was the exposure adequately ascertained?	1
	3. Was the outcome adequately ascertained?	1
Causality	4. Were other alternative causes that may explain the observation ruled out?	1
	5. Was there a challenge/rechallenge phenomenon?	0
	6. Was there a dose–response effect?	0
	7. Was follow-up long enough for outcomes to occur?	1
Reporting	8. Is the case(s) described with sufficient details to allow other investigators to replicate the research or to allow practitioners make inferences related to their own practice?	1
		5
